# Mouth and facial informativeness norms for 2276 English words

**DOI:** 10.3758/s13428-023-02216-z

**Published:** 2023-08-21

**Authors:** Anna Krason, Ye Zhang, Hillarie Man, Gabriella Vigliocco

**Affiliations:** Department of Experimental Psychology, University College London, 26 Bedford Way, London, WC1H, 0AP UK

**Keywords:** Mouth and facial movements, Visual informativeness, Word recognition

## Abstract

Mouth and facial movements are part and parcel of face-to-face communication. The primary way of assessing their role in speech perception has been by manipulating their presence (e.g., by blurring the area of a speaker’s lips) or by looking at how informative different mouth patterns are for the corresponding phonemes (or visemes; e.g., /b/ is visually more salient than /g/). However, moving beyond informativeness of single phonemes is challenging due to coarticulation and language variations (to name just a few factors). Here, we present mouth and facial informativeness (MaFI) for words, i.e., how visually informative words are based on their corresponding mouth and facial movements. MaFI was quantified for 2276 English words, varying in length, frequency, and age of acquisition, using phonological distance between a word and participants’ speechreading guesses. The results showed that MaFI norms capture well the dynamic nature of mouth and facial movements per word, with words containing phonemes with roundness and frontness features, as well as visemes characterized by lower lip tuck, lip rounding, and lip closure being visually more informative. We also showed that the more of these features there are in a word, the more informative it is based on mouth and facial movements. Finally, we demonstrated that the MaFI norms generalize across different variants of English language. The norms are freely accessible via Open Science Framework (https://osf.io/mna8j/) and can benefit any language researcher using audiovisual stimuli (e.g., to control for the effect of speech-linked mouth and facial movements).

## Introduction

The human face contains many important communicative cues that are part-and-parcel of face-to-face communication. These include movements of the mouth, cheeks, nose, eyebrows, and eye gaze (see Holler, 2022 for a recent review). Studies investigating audiovisual speech perception and comprehension most often manipulated the presence of these cues, by comparing performance on audiovisual (with visible mouth) and auditory-only stimuli (e.g., Ross et al., 2007; Tye-Murray, Sommers, & Spehar, 2007; Arnold & Hill, 2001), or by looking at performance in multimodal contexts (face only or face and body) in which the mouth area was blurred (e.g., Drijvers & Özyürek, 2017; IJsseldijk, 1992; Marassa & Lansing, 1995; Thomas & Jordan, 2004). However, removing certain features from the face and body lacks ecological validity, as it creates unnatural stimuli and does not inform about the effect of mouth and facial movements alongside other cues. In the current study, we describe a fine-grained method for obtaining mouth and facial informativeness (hereafter “MaFI”), i.e., how easy it is to identify English words based on their mouth and facial movement patterns, and report 2276 MaFI norms that capture and account for invariably present, but more or less informative, mouth and facial cues. MaFI norms can be used in studies employing more naturalistic stimuli where all communicative cues are available.

### Mouth and facial movements impact auditory perception

It is well established that mouth and facial movements impact auditory speech perception and comprehension (Bernstein, 2012; Peelle & Sommers, 2015). McGurk and MacDonald (1976) first demonstrated that attending to speech accompanied by incongruent mouth movements gives rise to an audiovisual illusion (for instance, presenting participants with an auditory signal “papa” that are visually similar on the mouth to “kaka” is perceived as a fused word “tata”), whereas Sumby and Pollack (1954) showed that seeing congruent mouth movements improves auditory speech processing in noise, and that this facilitatory effect increases as signal-to-noise ratio (SNR) decreases, i.e., when the speech becomes harder to understand. The benefit of audiovisual speech over auditory-only signal has been replicated in native speakers under both clear (Arnold & Hill, 2001; Reisberg et al., 1987) and noisy listening conditions (Ma et al., 2009; Ross et al., 2007; Schwartz, Berthommier, & Savariaux, 2004; Sumby & Pollack, 1954), as well as in non-native speakers (Hirata & Kelly, 2010; Drijvers & Özyürek, 2018; Drijvers et al., 2019), and it holds when looking at words (Sumby & Pollack, 1954; Ross et al., 2007), sentences (van Engen et al., 2017; Grant & Seitz, 2000) and discourse comprehension (Arnold & Hill, 2001; Reisberg et al., 1987).

One of the reasons why people benefit more from audiovisual speech relative to auditory-only is because (in particular) mouth movements inform about temporal and phonological processing that constrains phoneme identification (Peelle & Sommers, 2015). However, mouth and facial movements differ in how informative they are, as some are visually less ambiguous than others, and therefore can inform processing to different degrees. For example, consonants produced at labial (e.g., /b/, /p/, /m/) or labial-dental (e.g., /f/, /v/) positions (e.g., Binnie, Montgomery, & Jackson, 1974; Benguerel & Pichora-Fuller, 1982), as well as vowels with a rounding feature (e.g., /u/, /o/, /ɔ/; Robert-Ribes et al., 1998; Traunmüller & Öhrström, 2007) have long been recognized as visually more identifiable. Despite the robustness of the audiovisual enhancement effect, there is a large individual variability in how much people benefit from visual speech, and recent studies have suggested that it may depend on previous facial exposure (Rennig et al., 2020), as well as age and working memory (Schubotz et al., 2020).

### Measuring mouth and facial informativeness

Several measures of MaFI have been proposed. The most common is to measure speechreading performance in terms of overall percent accuracy or number of correctly guessed phonemes. Speechreading tasks often involve identifying silent phonemes in nonsense syllables, with the assumption that it informs about early word perception, or silent words embedded in sentences, which informs instead about how the perceptual information integrates with higher-order information, such as syntactic or semantic (Bernstein, 2012). Speechreading is, however, generally difficult with studies demonstrating an overall phoneme identification accuracy being often well below chance level (e.g., Bernstein et al., 1998; Fisher, 1968; Walden et al., 1981). One of the reasons why speechreading is so challenging is because some phonemes can easily be confused with others that are visually similar (Fisher, 1968). To account for visual confusions, researchers have analyzed the audiovisual transmission of articulatory features by creating confusion matrices of the responses and computing how much information for a particular feature is correctly processed by the perceiver (e.g., Iverson et al., 1997, 1998; Moradi et al., 2017). Such an approach is computationally heavy, and researchers generally agree on clustering visually similar phonemes into classes called visemes (Massaro, 1998; Fisher, 1968). For example, /b/ and /p/ belong to the same viseme class as they are hardly distinguishable from mouth movements alone in contrast to /b/ and /k/ that differ in how they are represented visually. Thus, there is no (or little) visual difference between phonemes within a viseme class, but viseme classes are meaningfully different from each other (Massaro et al., 2012). The borderline between viseme classes is, however, fuzzy, as it depends on factors such as speaker variability and phonetic context, i.e., the surrounding phonemes in a word, to name a few (Owens & Blazek, 1985).

To investigate viseme features that make words visually more salient, Jesse and Massaro (2010) presented a set of single-syllable words (CVC) in auditory-only, visual-only, and audiovisual conditions embedded in a gating task in which individuals had to identify words based on their onsets. The authors found that visemes with features including lower lip tuck (tucking the lower lip under the upper teeth, as in pronunciation of e.g., /v/), protrusion (sticking the lips out, e.g., /ʃ/), labial closure (sealing the upper and lower lips, e.g., /p/), mouth narrowing (horizontally bringing the lips closer, e.g., /w/), and finally rounding (creating a rounded shape with the lips, e.g., /r/) were visually more salient than others and improved word identification. Jesse and Massaro (2010) also investigated temporal distribution of the visual information and demonstrated that when it is available early during phoneme production (before the end of its first phoneme), it is particularly useful to auditory speech processing. This effect may be related to the fact that visual speech information precedes auditory signal by approximately 100–300 ms (Chandrasekaran et al., 2009; van Wassenhove et al., 2005), and thus, influence word recognition early by ruling out certain sounds and predicting others. Recently, Karas et al. (2019) found that words with a “visual head start” (in which the mouth movements begin significantly earlier than the auditory information, e.g., “drive” compared with “known”) showed a larger audiovisual benefit over auditory-only speech than words without a visual head start. This finding further suggests that the information from the mouth movements that occurs early in words is particularly useful and facilitates word recognition.

### Challenges of studying mouth and facial informativeness for words

Looking solely at the confusion matrix or mean number of correctly identified silent phonemes/visemes and visual information available early during word production (e.g., for words with a visual head start) may, however, be insufficient to capture the dynamic nature of mouth and facial movements for words (or sentences) as their informativeness can change depending on coarticulation, word length, and lexical similarity among other things. Let us look at the example: Is the word “moon” (/muːn/) more or less informative based on mouth and facial movements than the word “thermometer” (/θəʳmɒmɪtəʳ/)? The former starts with a visually salient labial movement (/m/), whereas the latter involves two such labial movements, but they occur later in the word (in the second and third syllables). “Thermometer” also contains other visually informative phonemes, e.g., tongue-tip movements and dental abduction of /θ/ and two rounding movements of /ʳ/, and has four times more syllables than the word “moon”. Longer words imply, as a matter of course, more mouth and facial movements. These, however, may either boost informativeness (if they contain enough visually salient information for the perceivers) or reduce it (if they cannot be easily identifiable and therefore become a distraction). As discussed earlier, the position effect (i.e., visually informative phonemes/visemes that are produced earlier show larger effect on auditory perception; Jesse & Massaro, 2010) also makes the comparison between “moon” and “thermometer” more challenging. That is, although both words contain a visually salient consonant /m/, they may not be equally informative as it appears in the initial position for “moon” but only later in the word “thermometer”. As most of the previous studies investigating visual saliency have used sets of words of a limited length or focused on word onsets (e.g., Auer Jr, 2009; Jesse & Massaro, 2010; Karas et al., 2019; Marassa & Lansing, 1995; Mattys et al., 2002), it is not clear whether and if so, then how visual information available later within a word also facilitates speech processing, which is particularly relevant for longer words (i.e., composed of two or more syllables).

Phonetic context and lexical distinctiveness also influence the informativeness of mouth and facial movements. Benguerel and Pichora-Fuller (1982) found that while speechreading performance of VCV syllables with visually more salient mouth movements (including articulation of /p/, /f/, /u/) was high regardless of the subsequent phonemes, phonetic context largely affected identification performance of mouth movements with lower visual saliency (as in articulation of /t/ or /k/). Indeed, the shape of the mouth during the execution of /t/ in “tick” (/tɪk/) and “talk” (/tɔ:k/) will be different because of the subsequent vowels that belong to distinct viseme classes (Massaro, 1998). Moreover, there is a lack of lexical distinctiveness between some phonemes. For instance, although /p/, /b/, and /m/ belong to the same viseme class, they would have a larger impact on intelligibility in a word such as “bat” than “bought” because of greater competition between lexically similar words (Auer, 2009; Auer & Bernstein, 1997, Mattys et al., 2002). That is, “pat” and “mat” are both compatible candidates in the first example, whereas “pought” and “mought” cannot act as lexically plausible candidates in the second example as they are not real words. Looking back at our example, “moon” and “thermometer” have substantially different phonetic contexts and lexical distinctiveness, which makes the comparison of their visual informativeness based on phonemes/visemes alone even more difficult, suggesting the need for a norming approach that will be useful to assess mouth and facial informativeness for words.

### Mouth and facial informativeness norms in behavioral and electrophysiological studies

Establishing MaFI norms for words could be useful in studies predicting behavioral and electrophysiological performance. Recently, MaFI norms (as described here) have been used to investigate multimodal (including face, hand gestures, and prosody) word and discourse comprehension and were found to be significant predictors (Krason et al., 2021; Zhang et al., 2021a, b). Krason et al. (2021) presented participants with pictures of everyday objects or actions followed by videos of a speaker uttering a word while producing a gesture that was either matching (i.e., it was imagistically related to the word uttered, as in moving a fist up and down while saying “hammer”) or mismatching (as in moving a fist up and down while saying “guitar”), or followed by a video with a still speaker saying the word. The authors also manipulated the clarity of the speech such that the words were either clearly audible or moderately noise-vocoded (using a six-band pass filter). Participants’ task was to judge whether the speech matched the pictures. MaFI norms were used instead of manipulating the presence of the lips to assess, in a more naturalistic way, the role of mouth and facial movements in multimodal speech comprehension. It was found that more informative mouth movements speeded up processing of the words across speech clarity conditions, but only in the absence of gestures. Further, when looking at speech accompanied by either matching or mismatching gestures, the authors found that individuals benefited from more informative mouth movements but only in the degraded speech conditions. Altogether, these findings suggest that people differentially weight the information from facial and limb movements and the use of a particular cue depends on its informativeness.

Across two EEG studies (Zhang et al., 2021a, b), Zhang and colleagues investigated how comprehenders (both native and non-native speakers) process multimodal passages input containing various cues, such as mouth movements, hand gestures, and prosody variations. The authors presented participants with videos of an actress acting short stories in a natural manner. The authors quantified the informativeness of each multimodal cue (also using the norms of informativeness presented here for mouth and facial movements), and then assessed the extent to which each cue (and the interactions between them) predicted the N400 amplitude – an EEG component peaking at ~ 400ms after word onset that has been associated to difficulties in processing (Kutas & Federmeier, 2011). For native speakers, the authors found that words with higher mouth informativeness elicited a less negative N400, in particular when they co-occurred with gestures, indicating that the more informative the mouth movements are, the more they facilitate language comprehension (Zhang et al., 2021a, b). A similar facilitatory effect of mouth informativeness was found for non-native speakers (Zhang et al., 2021a, b).

### The current study

Despite extensive work on the role of mouth and facial movements in auditory processing, the existing metrics for assessing visual informativeness may not capture the full complex dynamics of these movements, particularly when it comes to word-level processing. This limitation arises from various factors, including the influence of phonological context and co-articulation within a word, and the distinctive characteristics of individual words, such as word length, frequency, and age of acquisition, as discussed above. The current study addresses this gap by providing publicly available MaFI norms for 2276 English words, differing in their visual saliency, length, frequency, concreteness, and age of acquisition (AoA), together with videos of a speaker uttering those words. The paper comprises two parts. In the first part of the paper, we thoroughly describe the MaFI quantification that consists of measuring phonological distance between speechreading guesses and target words. We then report results from linear mixed effect regressions investigating features of phonemes and visemes that are good predictors of MaFI scores. We predict that MaFI scores will capture well the features that have been suggested to be visually more salient, such as frontness and roundness of phonemes, as well as lower lip tuck, lip closure, protrusion, and lip round (e.g., Jesse & Massaro, 2010). In the second part, we report results from correlation analyses. Here, we predict that MaFI scores will be highly correlated across different English variants, suggesting their generalizability.

## Part 1: Mouth and facial informativeness norms

In this section, a detailed description of MaFI for word quantification is provided. We also present results from confirmatory analyses investigating significant predictors, based on phoneme and viseme features, of MaFI norms.

## Methods

### Participants

Participants were 263 native British English speakers, and 147 native speakers of North American English with no language-related disorders or hearing difficulties. Participants were recruited from Prolific (http://www.prolific.co) on five different occasions: British participants were recruited for studies B1 (Experiment 1 from Zhang et al., 2021a), B2 (Experiment 2 from Zhang et al., 2021a), and B3, whereas American participants were recruited for studies A1 (Krason et al., 2021), and A2. Table 1 shows participants’ number and demographic information. The ethical approval was obtained from University College London (UCL; Research Ethics Committee 0143/003).

**Table 1:** Demographic information of participants

Study	Number of participants	Native tongue (English variant)	Mean age (SD)	Gender
B1 (Experiment 1 from Zhang et al., 2021a)	150	British English	28 (6.45)	F = 111 M = 37 Non-binary = 2
B2 (Experiment 2 from Zhang et al., 2021a)	59	British English	26 (7.13)	F = 40 M = 18 Non-binary = 1
B3	54	British English	29 (6.48)	F = 27 M = 27
A1 (Krason et al., 2021)	137*	American English	29 (6.24)	F = 71 M = 64 Non-binary = 2
A2	10	American English	30 (3.11)	F = 5 M = 5

*A total of 145 participants were tested, but eight were excluded due to technical errors or missing catch trials

### Materials

A total of 2544 words that varied in the number of phonemes (range 1–12), log-frequency (Balota et al., 2007, range 0–15.897), AoA (Kuperman et al. 2012, range 2.37–14.75; 276 words missing AoA norms) were video-recorded. These include 1678 words uttered by a native British English actress (B1-B3, with 100 words uttered twice) and 866 words uttered by a native American English actress (A1-A2). The same 168 words were produced by both actresses. The actresses were of similar age (late-20s/early-30s) and spoke with neutral accents and facial expressions. Each video (approx. mean length of 1 s) depicted the face of one of the actresses uttering a word. The videos were recorded with a professional camera (Panasonic HC-V180) either at UCL in a sound-proof recording booth (studies B1, A1) or at an actress’ home due to COVID-19 restrictions that were present in the United Kingdom at the time of stimuli preparation (studies B2, B3, and A2). The videos were muted for the purpose of the experiment.

### Procedure

**Fig. 1:**
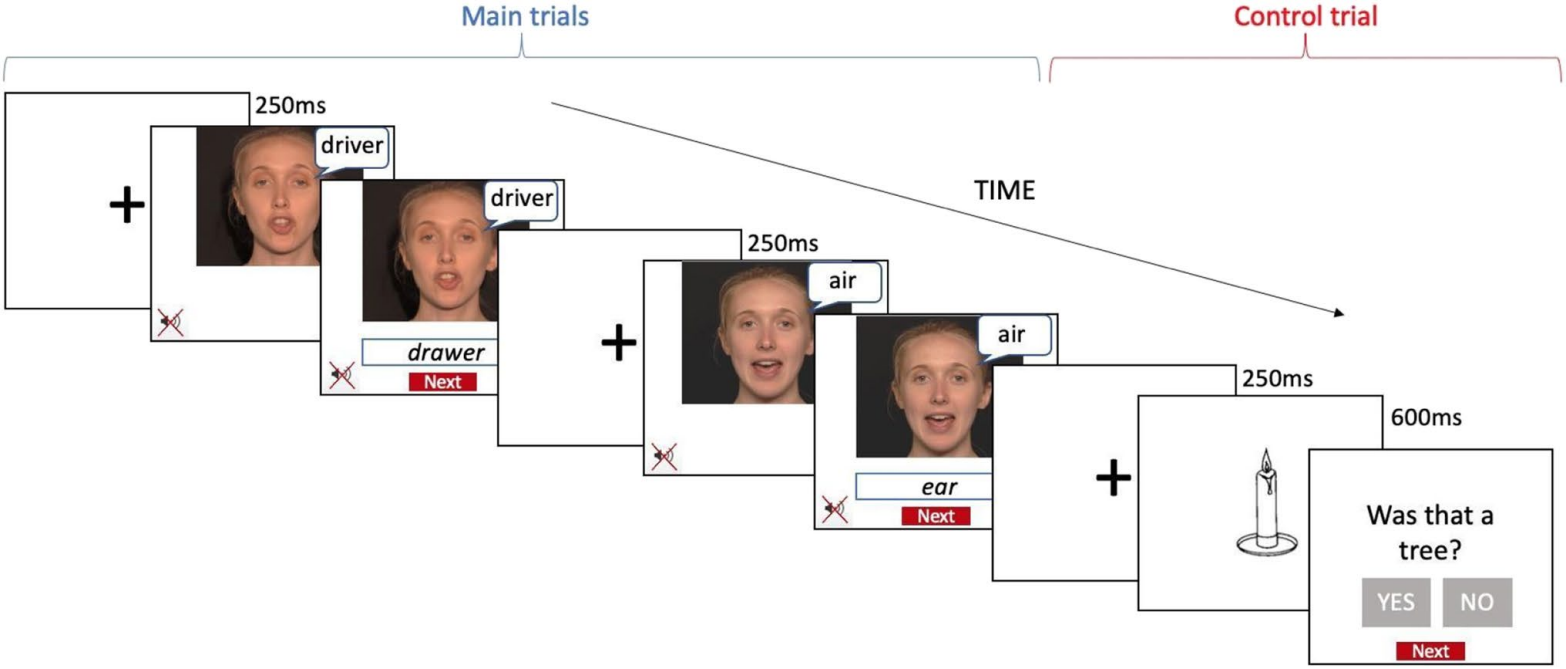
Example of two experimental trials and a control trial

Participants took part in an online experiment created on Gorilla (https://gorilla.sc/) that lasted between 20 and 40 min. Participants were only permitted to participate using a computer or laptop. Participants’ task was to watch muted videos and guess the word produced by the speaker by typing it in the answer box provided. Participants completed a list of randomly selected words from the entire corpus (60 words in study B1, 50 in B2, 120 in B3, 100 in A1, and 124 in A2). Each word was guessed by at least ten different participants. Participants initiated the videos by clicking on them and each video was automatically presented twice in a row to reduce task difficulty, and to make sure participants did not miss the beginning of each trial. A typing box appeared simultaneously with the second presentation of a video. The videos occupied two-thirds of the screen as depicted in Fig. 1. There was a 250-ms interval between the trials. Before the experiment, participants were exposed to seven trials followed by feedback for practice purposes and were encouraged to make their best guess if unsure of the correct answer. There were several self-paced breaks within the experiment to minimize fatigue. Additionally, we included 12 control trials, consisting of a lexical decision task where we showed participants pictures of everyday objects followed by a question (e.g., “Was this a tree?”). The control trials were randomly distributed within the experiment to identify participants who did not pay attention to the task. Performance on the control trials was above chance level in all the studies. Figure 1 depicts an example of trial types used in the experiment.

#### Mouth and facial informativeness quantification

To assess MaFI per word, we used a speechreading task, in which a fully visible face was presented to account for the information conveyed not only in visible mouth movements, but also in other orofacial movements, such as those of the jaw or cheeks, which have been shown to highly correlate with speech acoustics (Vatikiotis-Bateson et al., 1996; Yehia et al., 1997). A speechreading task, where participants had to type down their guesses, was chosen instead of a matching task (e.g., watching silent video-clips and guessing what was uttered by the speaker by choosing the correct answer among several foils) to avoid any effect of foil selection and ensure response variability between participants. The open format of the task has certain limitations. For instance, participants' responses might have been affected by lexical variables, which can lead to randomness in the results. To reduce the impact of random responses and control for lexical variables, we calculated the average scores across participants and added multiple control variables in our statistical models (see more details below). Moreover, speechreading words is often challenging due to insufficient visual information allowing word selection from the lexicon. Words with mouth and facial movements that convey too little information to correctly guess the words based on speechreading will therefore result in low MaFI.

After collecting the speechreading data, we calculated how similar (or distinct) are participants’ speechreading guesses to the target words. We first manually corrected accidental spaces and obvious typing errors (e.g., “barbeque” was corrected into “barbecue”) and removed any missing responses (1.3%). We then phonetically transcribed the target words and participants’ responses using a Python library *Epitran*^1^ (Mortensen et al., 2018). Next, to calculate feature-based string distance between the two words (a target and its corresponding response), we used *PanPhon*, which is a Python package with a database of over 5000 International Phonetic Alphabet (IPA) segments and their 21 phonological (articulatory) features (including sonorants, nasals, affricates etc.; for more information see Mortensen et al., 2016). Specifically, we measured the similarity of the phonological features of the IPA segments using the *“jt_weighted_feature_edit_distance_div_maxlen()”* function in *PanPhon*. The similarity is computed by calculating the number of string edits required to get from a participants’ response to the target word and dividing each score by the length of the longest word (either the target or the response word). Phonological features were weighted according to their phonological class and variability (see Mortensen et al., 2016) for justification and citations to other studies using a similar weighting method). The outcome of the string distance is a scalar variable with larger values signifying larger dissimilarity between the two linguistic units (here, words). 

Let's say that the target word is "bat" (/bæt/) and a participant provided the answer "pat" (/pæt/). The only difference between these two words is the voicing feature, which is assigned a weight of 0.125 by *PanPhon*. Since /b/ is voiced (+ 1) and /p/ is unvoiced (– 1), the cost of the change is equivalent to two units, each at a cost of 0.125. Therefore, the total edit cost is 2*0.125 = 0.25. To calculate the distance between the two words, the length of the longest word is considered, and the editing cost is divided by the number of phonemes in the longest word. In this case, the longest word has three phonemes, so the distance between "bat" and "pat" is 0.25 (editing cost) divided by 3 (longest number of phonemes) which equals 0.08. In comparison, if the target word were “bat” (/bæt/) and the participant had responded with “cat” (/kæt/), there would be five different features between the two words. The first feature is voicing, as /b/ is voiced (+ 1) and /k/ is unvoiced (– 1), which is assigned a weight of 0.125. Besides, /b/ and /k/ also differ in anterior, labial, high and back features, each weighted 0.25 according to *PanPhon*. Therefore, the editing cost is 2 *0.125 (voicing) + 2*0.25 (anterior) + 2*0.25 (labial) + 2*0.25 (high) + 2*0.25 (back) = 2.25. Again, the editing cost is divided by the number of phonemes in the longest word (here 3), resulting in a score of 0.75. Thus, “bat” and “pat” are more similar to each other (as the distance value is closer to 0) compared to “bat” and “cat” (as the distance value is 0.75). After calculating the distance values for each target word individually for every participant, we computed the average distance value per word, by taking the mean of the distance values obtained from each participant and multiplied it by – 1 for ease of interpretation of the results. That is, a score close to 0 indicates small distance/highly informative mouth and facial movements, and the more negative the score is, the less informative the movements are, which hereafter is called “MaFI scores”.

**Fig. 2:**
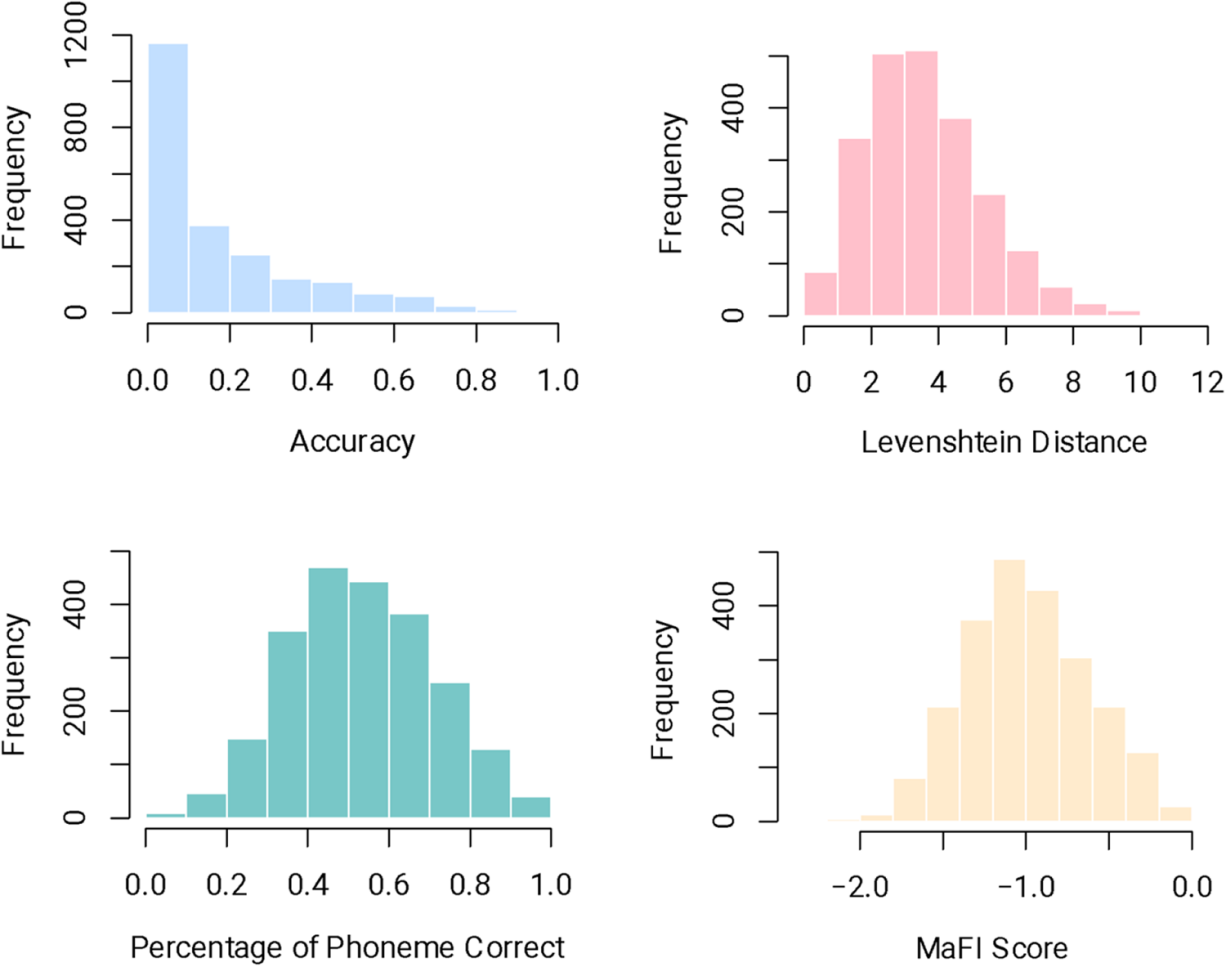
Distribution of speech reading accuracy, Levenshtein distance, the percentage of phoneme correct, and MaFI scores for all words

Finally, we calculated the Levenshtein distance (which is a measure of (dis)similarity between two strings of characters based on the number of edits, such as deletions, insertions, and substitutions, needed to transform from one string to another; Levenshtein, 1965), mean speechreading accuracy, as well as mean percentage of phoneme correct^2^ per each word for comparison (see Fig. 2). We also calculated correlation coefficients of these other measures with MaFI scores. First, we found that the distribution of the accuracy and Levenshtein distance data is skewed, making them less suitable for analysis assuming normality. While both the percentage of phoneme correct and MaFI score are normally distributed, we show that our norms better capture the similarity between participants’ responses and the target word. Assuming that the target word was “bat” (/bæt/), and one of the responses was “cat” (/kæt/) while the other was “pat” (/pæt/), the mean accuracy of correctly identified phonemes is 0.66 in both cases. However, this score does not account for the fact that “pat” (/pæt/) is hardly indistinguishable from “bat” (/bæt/) in the visual context. Our MaFI scores, instead, are different for the two words, with “cat” (/kæt/) being – 0.75 and “pat” (/pæt/) being – 0.08, demonstrating that “pat” (/pæt/) is visually more similar to “bat” (/bæt/). Second, we calculated the correlation between MaFI scores and the mean speechreading accuracy (*r* = 0.74, *p* < 0.001), mean percentage of phoneme correct (r = 0.87, *p* < 0.001) and the Levenshtein distance (*r* = – 0.70, *p* < 0.001, note that the correlation is negative because a larger Levenshtein distance indicates less accurate responses). The correlation is high in all cases, suggesting the validity of our norms.

The MaFI norms are publicly available on the OSF repository (https://osf.io/mna8j/) and will be extended with new informativeness scores in the future. Other researchers are welcome to collaborate with us on expanding the corpus. The repository includes:

i) The averaged MaFI scores for 2276 English words together with their unaveraged raw scores. The scores are presented as a whole corpus (including responses collected across all studies; note that the scores for duplicate words were averaged) and separately for the studies with British (B1-B3) and American (A1-A2) speakers.
ii) Mean accuracy, the percentage and number of correctly identified phonemes, as well as Levenshtein distance per word (averaged and unaveraged).
iii) Words’ lexical and phonetic features that were used in the analyses (see sections below).
iv) Video-stimuli of British and American actresses producing isolated words.

## Data analysis and results

We performed feature analyses (separately on phonemes and visemes categories based on Jesse & Massaro, 2010) for all words^3^ using hierarchical multiple linear regressions with MaFI scores as our dependent variable. The predictors of interest included:

- For the phoneme feature analysis: frontness and roundness. Words including a front place of articulation, including bilabials (/b/, /p/, /m/) and labial-dentals (/f/, /v/; e.g., Binnie et al., 1974; Benguerel & Pichora-Fuller, 1982; Jesse & Massaro, 2010), as well as phonemes with a rounding feature (/r/, /w/, /u/, /o/, /ɔ/; Robert-Ribes et al., 1998; Traunmüller & Öhrström, 2007; Jesse & Massaro, 2010) should be visually more salient as indicated by higher MaFI scores. As a confirmatory analysis, we also calculated the “informativeness load” for each word to assess whether the number of informative phonemic features significantly predicts MaFI scores. This was done by counting the number of front and rounded phonemes per word and dividing it by the total number of phonemes in that word.
- For the viseme feature analysis: lower lip tuck (viseme {f}, including phoneme /f/, /v/), protrusion (viseme {ch}, including phoneme /ʃ/, /tʃ/, /dʒ/, viseme {w}, including phoneme /w/), labial closure (viseme {p}, including phoneme /b/, /p/, /m/), and lip rounding (viseme {j}, including phoneme /j/, viseme {r}, including phoneme /r/, and viseme {w}, including phoneme /w/). Words including these visemes should be visually more salient (Jesse & Massaro, 2010) as indicated by higher MaFI norms. Similarly to phoneme feature analysis, we also calculated the informativeness load per word based on the informative viseme features by counting the number of visemes with lower lip tuck, protrusion, labial closure, and lip rounding features and dividing it by the total number of visemes in that word.

Each target model described above included several lexical variables, such as number of phonemes, AoA (Kuperman, Stadthagen-Gonzalez, & Brysbaert, 2012), log-frequency (Balota et al., 2007), phonological neighborhood density (Luce & Pisoni, 1998)^4^ to obtain effects of phoneme and viseme features while controlling for lexical variables statistically. We also fitted a baseline model that included lexical variables alone. We compared the target models with the baseline model with the log-likelihood method, using the anova() function built in R. Significant improvements of the target models would indicate that phoneme/viseme features contribute to MaFI above and beyond lexical features. All categorical variables were dummy coded (so that we compared words with specific features to words without these features), and the continuous variables were scaled. The analyses were carried out in RStudio (V. 4.0.4), and the R code is available on OSF (https://osf.io/mna8j/).

**Fig. 3:**
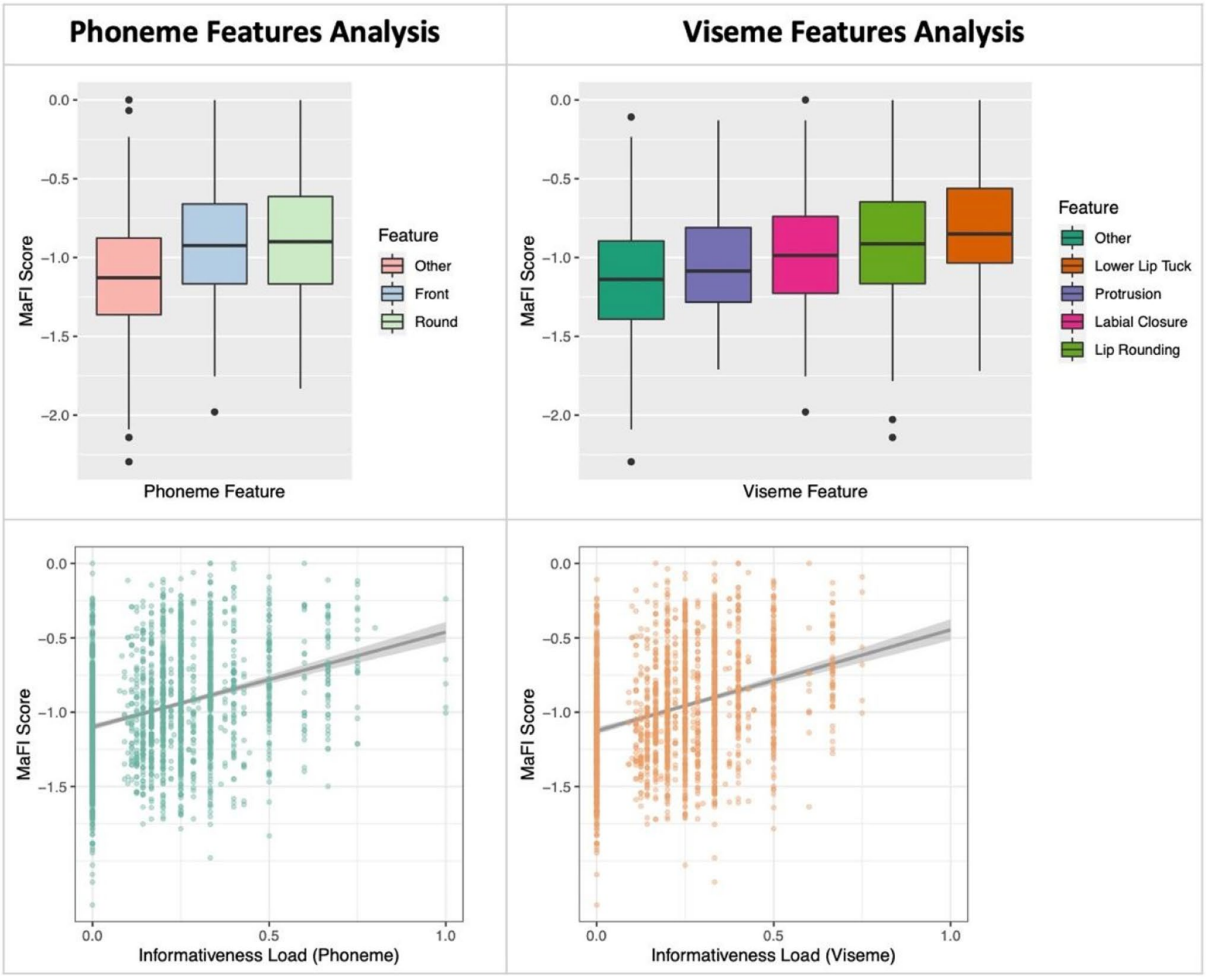
Mean MaFI scores for phoneme and viseme features and their informativeness load. *Note:* “Other” represents the intercept (i.e., reference level) and refers to all words that do not contain the informative features

The target models with additional phoneme/viseme features showed significant improvements compared with the baseline model (*p* < 0.001 in all cases), and thus, the results reported next will refer to the target models only. We found that words containing phonemes with roundness and frontness features (i) as well as words containing visemes characterized by lower lip tuck, protrusion, labial closure, and lip rounding (ii) had overall a higher MaFI score, indicating that these words are visually more informative. As predicted, we also found that larger informativeness load (both for phonemes and visemes) led to higher MaFI score, indicating that words with a larger proportion of informative features have more informative mouth and facial movements. The full set of results is presented in Table 2. Figure 3 depicts mean informativeness scores with informative features for phonemes with their informativeness load (left panel) and visemes with their informativeness load (right panel). In conclusion, these results show that the presence of certain phonemes and visemes, particularly those with roundness and frontness features, as well as a larger proportion of informative features, contribute to a higher MaFI score, indicating that such words are visually more informative, which is in line with current literature on mouth and facial movements saliency. While lexical features such as frequency, age of acquisition and number of phonemes affects MaFI score, the phonetic and viseme features contribute to the visual informativeness of words beyond these lexical variables.

**Table 2:** Results of the feature analyses

**Phoneme feature analysis** (*R*^2^ = 0.15)					
	β	SE	***t***	***p***	
(Intercept)	– 0.37	0.03	– 11.01	< 0.001	***
Rounding	0.43	0.04	9.68	< 0.001	***
Frontness	0.50	0.04	12.06	< 0.001	***
Frequency	0.22	0.02	9.01	< 0.001	***
PhonNeighborhood	– 0.03	0.03	– 1.09	0.27	
AoA	– 0.06	0.02	– 2.56	0.01	*
PhonemeNumber	– 0.10	0.03	– 3.12	0.002	**
**Phoneme informativeness load** (*R*^2^ = 0.15)					
	β	SE	***t***	***p***	
(Intercept)	0.02	0.02	1.04	0.30	
InformativenessLoad_Phoneme	0.29	0.02	14.69	< 0.001	***
Frequency	0.21	0.02	8.63	< 0.001	***
PhonNeighborhood	– 0.05	0.03	– 1.91	0.06	
AoA	– 0.07	0.02	– 3.09	0.002	**
PhonemeNumber	0.00	0.03	0.06	0.95	
**Viseme feature analysis** (*R*^2^ = 0.19)					
	β	SE	***t***	***p***	
(Intercept)	– 0.42	0.03	– 12.13	< 0.001	***
LowerLipTuck	0.77	0.06	13.75	< 0.001	***
Protrusion	0.35	0.05	7.24	< 0.001	***
LabialClosure	0.37	0.04	8.65	< 0.001	***
LipRounding	0.19	0.04	4.29	< 0.001	***
Frequency	0.21	0.02	8.88	< 0.001	***
PhonNeighborhood	– 0.02	0.03	– 0.69	0.49	
AoA	– 0.09	0.02	– 3.70	< 0.001	***
PhonemeNumber	– 0.10	0.03	– 3.19	0.001	**
**Viseme informativeness load** (*R*^2 ^= 0.15)					
	β	SE	***t***	***p***	
(Intercept)	0.02	0.02	1.17	0.24	
InformativenessLoad_Viseme	0.30	0.02	15.02	< 0.00	***
Frequency	0.22	0.02	8.97	< 0.001	***
PhonNeighborhood	– 0.05	0.03	– 1.71	0.09	
AoA	– 0.09	0.02	– 3.93	< 0.001	***
PhonemeNumber	0.03	0.03	0.89	0.37

## Part 2: Generalizability of the norms

In this section, we investigate whether the MaFI norms generalize across different English language variants. To answer this question, we carried out two online experiments, in which participants had to speech-read words. In the first experiment, we asked speakers of four different English language variants (British, American, Canadian, and Australian) to each produce and video-record 100 words that were then watched by participants of the same language background (hereafter “perceivers”). The goal of this experiment was to assess whether MaFI of a certain word is similar when produced by speakers of different variants of English. If this is the case, then the MaFI norms should generalize across speakers of different English variants. In the second experiment, we compared the speechreading performance of participants of British English watching videos produced by an American speaker (the same actress as in the videos collected as a part of studies A1-A2) and compared it with the responses to the same videos by American participants (taken from studies A1-A2). Similarly, we compared American English participants’ performance in speechreading words produced by the British actress with the performance of British English participants (taken from studies B1-B3). The goal of this experiment was to assess whether MaFI norms were correlated when perceivers of different English-language variants watched the same word.

## Methods

### Participants

A total of 100 participants were recruited from Prolific (http://www.prolific.co) to take part in two online experiments created on Gorilla (https://gorilla.sc/). For Experiment 1, we recruited 20 native speakers of British English, 20 native speakers of American English, 20 native speakers of Canadian English, and 20 native speakers of Australian English. For Experiment 2, we additionally recruited ten speakers of British English and ten speakers of American English. As in the experiments in Part 1, each word was answered by at least ten participants. The experiments were conducted under the same UCL ethical approval as in Part 1 (Research Ethics Committee 0143/003). Table 3 presents participants’ demographic data.

**Table 3:** Participants’ demographic information for Experiments 1 and 2

Exp.	Number of participants	Participants’ native tongue (English variant)	Mean age (SD)	Gender
1	20	British English	28 (4.84)	F = 10, M = 10
1	20	American English	28 (5.65)	F = 11, M = 9
1	20	Canadian English	31 (6.51)	F = 10, M = 10
1	20	Australian English	30 (5.39)	F = 10, M = 10
2	10	American English	29 (5.50)	F = 5, M = 5
2	10	British English	25 (6.07)	F = 5, M = 5

### Materials

The materials used for Experiments 1 and 2 contained the most frequent 100 words selected from our corpus. In Experiment 1, we asked eight monolingual speakers of different English variants (including British, American, Canadian, and Australian; mean age = 33, SD = 4.65) to each produce, as naturally as possible, all 100 words. One female and one male speaker were included per language variant. The words were video recorded using different devices (i.e., either a video-recorded or a phone camera) at speakers’ homes. The videos were then edited by the researchers mimicking the procedure described in Part 1, i.e., the videos were cropped, so that only the face of the speaker was in view and the audio was muted. For Experiment 2, we used the recordings produced by the British and American actresses prepared for the B1-B3 and A1-A2 studies described earlier.

### Procedure

Experiments 1 and 2 adopted the same procedure as described earlier for the MaFI experiment (see Part 1 for more information), with the task to guess, by typing down the answers in a box provided, words silently uttered by a speaker in the videos. In Experiment 1, participants were assigned to guess 100 words produced by a speaker of the same English variant (e.g., Canadian participants watched videos of a Canadian speaker). This was counter-balanced by gender, i.e., half of the words were produced by a female speaker and the other half of the words were produced by a male speaker. Each word was, therefore, guessed by 20 different participants (ten participants per female speaker and ten participants per male speaker). Experiment 2 was a cross-language variant experiment, where British participants watched 100 videos of an American English speaker producing words, and American participants watched 100 videos of a British English speaker.

## Data analysis and results

All 100 words were first transcribed into their IPA using *Epitran*, followed by MaFI score calculation that mimicked the procedure described in Part 1. All the analyses were performed in RStudio (V. 4.0.4).

**Fig. 4:**
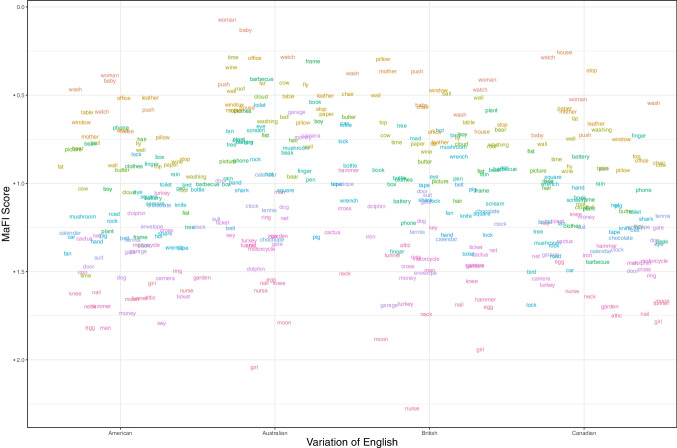
Mean MaFI scores for 100 words in four variants of English. Each word is represented by a distinct color, with the colors being consistent for the same words across all English variants

In Experiment 1, MaFI score for each word in each language variant was calculated averaging across the two speakers of the same English variant. The analysis for Experiment 1 consisted of a series of Pearson’s correlations, i.e., we correlated MaFI scores of the 100 words obtained from speakers of four different English variants (British, American, Canadian, Australian). All correlations showed high Pearson’s *r* (> 0.67), suggesting that MaFI norms are relatively consistent across speakers of different English variants. The results are presented in Fig. 4 and Table 4.

**Table 4:** Results of Pearson’s correlation between MaFI scores obtained by speakers of different English variants

English variant	Pearson’s correlation results
British & American	*r*(98) = 0.73, *p* < 0.001, 95% CI [0.63–0.81]
British & Australian	*r*(98) = 0.74, *p* < 0.001, 95% CI [0.64–0.82]
British & Canadian	*r*(98) = 0.71, *p* < 0.001, 95% CI [0.59–0.79]
American & Australian	*r*(98) = 0.69, *p* < 0.001, 95% CI [0.58–0.78]
American & Canadian	*r*(98) = 0.70, *p* < 0.001, 95% CI [0.59–0.79]
Australian & Canadian	*r*(98) = 0.67, *p* < 0.001, 95% CI [0.54–0.76]

**Fig. 5:**
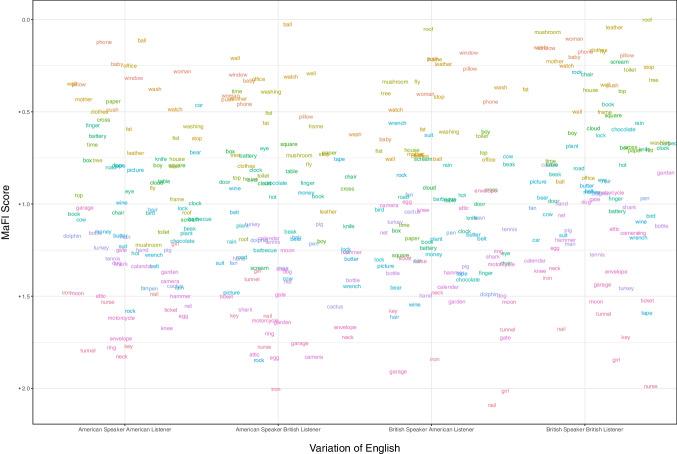
Mean MaFI scores for 100 words produced by an American or British speaker and guessed by American or British participants. Each word is represented by a distinct color, with the colors being consistent for the same words across all English variants

In Experiment 2, we investigated the effect of cross-language variation on the MaFI scores. The 100 words produced by an American actress were presented to British English participants, and their responses were compared with the responses of American participants watching the same videos, by calculating Pearson’s correlations between the two sets of MaFI scores. Similarly, the 100 words produced by a British actress were presented to American English participants, and we calculated the correlation of their MaFI scores with the responses from British participants. Again, all correlations showed high Pearson’s *r* (>0.74), suggesting that the relative informativeness of words (measured by MaFI scores) is highly similar when viewed by perceivers of different English variants. The results are presented in Fig. 5 and in Table 5.

**Table 5:** Results of Pearson’s correlation between MaFI scores obtained by perceivers of different English variants watching either an American or British actress silently producing words

Speaker’s English variant	Perceivers’ English variant	Pearson’s correlation results
American	British & American	*r*(98) = 0.83, *p* < 0.001, 95% CI [0.75–0.88]
British	British & American	*r*(98) = 0.74, *p* < 0.001, 95% CI [0.64–0.82]

## Discussion

In this article, we present a corpus of 2276 mouth and facial informativeness (MaFI) norms, i.e., quantification of how visually informative words are based on the corresponding mouth and facial movements. We first investigated the relationship between MaFI scores and visually salient features (e.g., bilabials), and found that MaFI scores capture well the informativeness of different phoneme and viseme features. Second, we tested the generalizability of the norms by comparing the MaFI scores obtained from speakers and perceivers across different English language variants, and we found that the MaFI scores are highly correlated regardless of language variant. Therefore, the MaFI scores can be used as a basic behavioral measure of visual speech informativeness across speakers and English variants. The norms are publicly available on the OSF repository (https://osf.io/mna8j/) that includes rescaled MaFI scores with their raw phonological distance scores, mean accuracy and percentage of number of correctly identified phonemes per word, as well as Levenshtein distance for comparison, lexical and phonetic features of words, and finally, the videos of British and American actresses uttering isolated words that were used in the studies described here. We invite other researchers to collaborate in the future on expanding the corpus.

### Mouth and facial informativeness norms as a valid behavioral measure

Mouth and facial movements are part of face-to-face communication, and they inform comprehenders to a different extent depending on their visual saliency. Our study provides a novel corpus of norms that quantify informativeness of these movements per word. Our findings from the feature analyses on the norms are in line with previous research focusing on individual phonemes and groups of visually indistinguishable phonemes, i.e., visemes. Specifically, we have shown that the presence of phonemes with frontness and rounding features (/b/, /p/, /m/, /f/, /v/, and /r/, /w/, /u/, /o/, /ɔ/, respectively) or visemes characterized by lower lip tuck ({f}), labial closure ({p}), protrusion ({ch}, {w}) and lip rounding ({j}, {r}, {w}) make the words more informative based on the corresponding mouth and facial movements, in line with previous studies (e.g., Binnie et al., 1974; Benguerel & Pichora Fuller, 1982; Robert-Ribes et al., 1998; Traunmüller & Öhrström, 2007; Jesse & Massaro, 2010). The analysis of the informativeness load (i.e., the number of informative features per word given its length) also supports these conclusions by showing that the more informative features a word contains (regardless of their position within a word), the more informative it becomes based on the corresponding mouth and facial movements. For instance, according to our norms, words such as “change”, “love”, “remove”, “officer”, and “mouthwash” are highly informative based on mouth and facial cues (all have a MaFI score above – 0.10) and can inform a perceiver to a larger extent than words such as, e.g., “needle”, “oxygen”, “mug”, “leek”, and “gorilla” (all have a MaFI score below – 1.7). Regarding our earlier examples of “thermometer” and “moon” that differed in word length, number of informative features, and their position within a word, “thermometer” is visually more informative than “moon” (– 0.75 versus – 1.50), but neither of these words seem to be on one of the extremes of our norms. Altogether, these results suggest that our quantification captures visual informativeness well for both shorter and longer words (composed up to five syllables).

Furthermore, given that our MaFI measure is based on silent speechreading, one can argue that the method is prone to large individual variability in speechreading skills as well as differences in pronunciation, not only across English languages, but also more generally across speakers. Here, we have shown that the MaFI scores obtained for speakers of different English language variants (including British, American, Canadian, and Australian) are highly correlated. Moreover, the norms generalize across perceivers of different English variants (at least for British and American perceivers), suggesting their usability. That is, the norms show that the relative informativeness of mouth and facial movement is (at least partially) specific to individual words and is consistent across different variants of English. Further research is, however, needed to investigate more thoroughly speaker-related differences, which is beyond the scope of the present study. In addition, the speechreading task used in the study was not limited to pure visual information, but was also influenced by other lexical factors. That is, we found that words that are used more frequently and acquired earlier in life also predict MaFI scores. This is not surprising since visual informativeness was evaluated on a word-by-word basis and thus was influenced by the words' lexical features. Other factors, such as word length and phonological neighborhood density, were also examined, but did not have a significant impact on the results, except for word length in the phoneme feature analysis. However, it is important to note that the MaFI score is not solely driven by the lexical features, as phoneme and viseme features showed significant effect after controlling for several lexical variables, and the models with phoneme and viseme features showed significantly better fit to the MaFI score than the baseline model with only lexical factors. Moreover, there were high frequency words with low MaFI scores (e.g., “girl”) and low frequency words with high MaFI scores (e.g., “leather”). Furthermore, previous studies have shown that MaFI scores still explain additional variance in audiovisual speech recognition even when controlling for lexical variables, including word predictability, frequency, and age of acquisition (Krason et al., 2021; Zhang et al., 2021a, b).

Finally, studies that have already employed MaFI norms show that they are a psychologically valid operationalization of the information provided by the face during not only word (Krason et al., 2021), but also discourse comprehension (Zhang et al., 2021a, b). MaFI norms also come with several key advantages and complements previous measures. For instance, given that individual words have their unique combination of sounds and mouth patterns, it is more ecologically valid to look at informativeness of the whole words rather than single phonemes, which are often pronounced differently based on phonological (e.g., Benguerel & Pichora-Fuller, 1982) and lexical (e.g., Auer, 2009) contexts. Measuring visual informativeness of a word based on informativeness of single phonemes/visemes further poses several issues as already discussed with the examples of “thermometer” and “moon”. Moreover, words are a common unit in experimental designs and psycholinguistic analysis and therefore it is useful to have MaFI norms at this level. Altogether, these norms can benefit any study investigating audiovisual (or visual) speech processing by quantitatively assessing the magnitude of the impact of mouth movements without the need to manipulate their presence versus absence. Given their continuous nature, mouth informativeness scores can be easily incorporated as a fixed or control variable in statistical models.

## Conclusion

To sum up, the norms presented here are a valid and fine-grained method of assessing the informativeness of different mouth and facial movements of isolated words. The norms are scalable as they (i) consider a range of words with different frequency, length, age of acquisition, as well as phonological neighborhood density; (ii) capture well phoneme and viseme features known to be visually salient; (iii) generalize across English language variants; iv) have been previously successful at predicting the impact of mouth and facial movements in behavioral and electrophysiological studies. The norms are publicly available on Open Science Framework (https://osf.io/mna8j/).

## Data Availability

Mouth and Facial Informativeness norms, the corresponding videos, as well as the analysed datasets during the current study are available at https://osf.io/mna8j/

## Footnotes

1 We also tested for the effect of different IPA transcriptions on the MaFI scores, by transcribing all words into IPA according to the speaker’s English-language variant (using https://tophonetics.com) and calculating MaFI scores anew. We then carried out Pearson’s correlations between these scores and the scores obtained based on Epitran transcriptions. The Pearson’s correlation coefficients were very high in all cases (*r*’s > 0.96).

2 We did not calculate mean viseme accuracy due to discrepancies between viseme classifications (particularly for vowels).

3 We also performed feature analyses separately for the words collected as a part of studies with British participants (B1-B3) and American participants (A1-A2). The results are consistent across all analyses and can be found on OSF for comparison.

4 We initially included orthographical neighbourhood density as a control variable but due to correlation with phonological neighbourhood density it was removed.

## Code availability

The code is available at https://osf.io/mna8j/
